# Tolerability and outcomes of radiotherapy or chemoradiotherapy for rectal cancer in elderly patients aged 70 years and older

**DOI:** 10.1186/1748-717X-8-86

**Published:** 2013-04-10

**Authors:** Xin Cai, Hongbin Wu, Junjie Peng, Ji Zhu, Sanjun Cai, Gang Cai, Zhen Zhang

**Affiliations:** 1 Department of Radiological Oncology, Fudan University Shanghai Cancer Center, 270 Dong'An Road, Shanghai, 200032, People’s Republic of China; 2 Department of Oncology, Shanghai Medical College, Fudan University, Shanghai, People’s Republic of China; 3 Department of Colorectal Surgery, Fudan University Shanghai Cancer Center, Shanghai, People’s Republic of China

**Keywords:** Rectal cancer, Elderly, Radiotherapy, Comorbidity, Outcome

## Abstract

**Purpose:**

To assess the safety and outcomes of radiotherapy (RT) or chemoradiotherapy (CRT) in elderly patients (≥70) with rectal cancer.

**Methods:**

Elderly patients aged 70 and older with rectal cancer, who were treated with RT or CRT at a single institution, were retrospectively analyzed. Performance status (KPS and ECOG score) and comorbidity (Charlson comorbidity index) were calculated, and their correlation with treatment toxicity and overall survival were studied. Risk factors for overall survival were investigated using univariate and multivariate survival analysis.

**Results:**

A total of 126 patients with locally advanced disease, local recurrence or synchronous metastasis were included, with a 3-year OS rate of 48.1%. Scheduled dosage of radiation was delivered to 69% of patients. Grade 3 toxicities occurred more often in patients treated with CRT versus RT. The occurrence of grade 3 toxicities was not related to KPS score, ECOG score, number of comorbidities, and Charlson score. Multivariate analysis found that only age and Charlson score were independent prognostic factors for predicting patients’ 3-year OS. The 3-year OS rate was significantly higher in patients with Charlson score <4 vs Charlson score ≥4 (71.1% vs. 26.4%, P=0.0003).

**Conclusions:**

Although toxicities may be significant, elderly patients with rectal cancer of varied stages can be safely treated with RT or CRT with careful monitoring and frequent modification of treatment. Except for patients’ age, Charlson comorbidity index may be helpful in assessing patients’ outcomes in elderly patients with rectal cancer.

## Background

Colorectal cancer (CRC) is the fifth most common malignancy in China. As the population ages, more and more elderly patients are diagnosed with CRC. In Shanghai, one of the most developed areas of China, patients older than 70 years accounted for 57% of all newly diagnosed colorectal cancers in 2006, compared with only 32% in 1990 [1]. Determining the optimal treatment for elderly patients with CRC is a complex process, especially in rectal cancer. Elderly patients are more likely to have other concomitant chronic illnesses (comorbidity), which may increase the risk of complications and even death during treatment. When treating elderly patients with rectal cancer, it is vital to determine how aggressively to treat, so that the costs and risks of the treatment will not outweigh the short-term benefits from treatment of the cancer. Recent studies have confirmed the survival benefit of adjuvant chemotherapy in elderly patients (≥75 years) with resected colon cancer [2]. However, the value of chemoradiotherapy (CRT) or radiotherapy (RT) in elderly patients with rectal cancer is still controversial. In addition to patients’ comorbidity, several factors including the higher morbidity rate of rectal cancer surgeries, stoma related complications, and the toxicity of RT or CRT, are also responsible for the limited use of standard treatment in elderly patients with rectal cancer.

Several scaling systems have been widely used to evaluate the outcomes and safety of treatment in elderly patients, including patients’ performance status (Karnofsky Performance Status Score [3] and ECOG Performance Status Score [4]) and comorbid conditions (Charlson comorbidity index [5]). The purpose of the current study was to assess the safety of RT or chemoradiotherapy in elderly patients (≥70 years) with advanced rectal cancer, and to evaluate the value of comorbidity index in predicting patients’ survival.

## Material and methods

### Patient and treatment protocol

A retrospective study was performed. We reviewed the hospital records of consecutive patients aged 70 years and older with rectal cancer who were treated in the Department of Radiological Oncology, Fudan University Shanghai Cancer Center between January 2002 and December 2010. All patients had histologically confirmed adenocarcinoma, and all tumors were located <12 cm from the anal verge as confirmed by colonoscopy or digital examination.

In our series, the patients could be categorized into 3 groups according to their treatment purpose: 1) Curative intent treatment: patients undergoing surgical resection of primary tumor with neoadjuvant or adjuvant RT or CRT; 2) Local control: patients with local recurrence or major comorbidity that were unsuitable for surgical resection; 3) Symptom control: patients with symptomatic primary tumor and unresectable synchronous distant metastases.

The treatment strategies and dosage of RT or chemotherapy were determined by comprehensive assessment of patients' tumor stage, age, performance status, comorbidity, and history of medication. RT treatments were performed according to institutional protocols. Conventional RT was used in all of the patients. The mean total dose of RT was 52.2Gy (50.4Gy in curative intent group, 53.8Gy in local control group, 52.6Gy in symptom control group), ranging from 30Gy to 66Gy.

### Treatment toxicity, performance status and comorbidity

During treatment, patients were monitored weekly for signs of acute toxic effects, with corresponding adjustments in chemotherapy regimens and RT protocols. Acute treatment-related toxicities were evaluated using the National Cancer Institute Common Toxicity Criteria for Adverse Events (CTCAE v3.0) [6]. Diarrhea, skin toxicity and neutropenia were the major evaluated effects in the current study.

Patients’ performance status was assessed by Karnofsky Performance Status Score (KPS Score) [3] and ECOG Performance Status Score (ECOG Score) [4]. Patients’ comorbidity was rated according to Charlson Comorbidity Index Score (Charlson Score) [5]. As the stage of primary tumor varied from stage I to stage IV in our series, the scores of baseline cancer disease were also calculated in Charlson score. The scoring algorithm for cancer is as follows: two points were scored in patients with primary tumor in situ, zero points were scored when primary tumor was surgically resected, and six points were scored in patients with distant metastases. As Charlson score does not specify the point score for local recurrence in rectal cancer, 6 points were scored for local recurrence because of similar overall survival compared with patients with distant metastases in our series (shown in Results).

### Statistics

Chi-square test and *t*-test were used to compare the rates of early toxicity between different treatment modalities, performance status (KPS and ECOG score) and comorbidities (Charlson Score). Three-year survival rate was used as the endpoint in outcome analyses. The Kaplan-Meier method was used to calculate overall survival rates with the log-rank test. Cox proportional hazards regression was used in univariate and multivariate analyses. P<0.05 was considered statistical significant.

## Results

A total of 126 patients aged 70 years and older were included in current study. The median age was 75 years old (range 70–92 years), with 58% of patients aged 75 years and older. 51 cases (40.5%) were treated with curative intent by surgical resection with adjuvant or neoajuvant RT/CRT. 47 patients (37.3%) were treated for local control, of which 33 patients were radiated due to local recurrence, and the other 14 patients were radiated alone due to major comorbidities. Out of 73 patients with concurrent CRT, 37 patients were treated with combination chemotherapy and 36 patients had single agent chemotherapy with 5-FU or capecitabine. Patients’ clinicopathological characteristics are listed in Table 1.

**Table 1:** Clinicopathological features of 126 elderly patients with rectal cancer

**Characteristics**		**No. (n=126)**	**%**
Age			
	70–74	53	42.1
	75–79	50	39.7
	>/=80	23	18.2
Gender			
	Male	84	66.7
	Female	42	33.3
Disease			
	Primary only	65	51.6
	Local recurrence	33	26.2
	Primary+Distant metastases	28	22.2
KPS Score			
	≥90	44	34.9
	≥80–<90	70	55.6
	<80	12	9.5
ECOG Score			
	0	22	17.5
	1	78	61.9
	2–3	26	20.6
Charlson Score			
	0–1	41	32.6
	2–4	23	18.2
	5–8	62	49.2
Comorbidities			
	None	72	57.1
	1	25	19.8
	2	22	17.5
	3–4	7	5.6
Treatment purpose			
	Curative Intent Treatment	51	40.5
	Local control	47	37.3
	Symptom control	28	22.2
Modality of RT			
	RT only	53	42.1
	CRT	73	57.9

*abbreviation: RT, Radiotherapy; CRT, Chemoradiotherapy.

### Performance status, comorbidity and early toxicity

Planned dosage of RT was delivered to 69% of patients. Dose modification or discontinuation of chemotherapy was required in 37.3% of patients. None of the patients in our series had grade 4 toxicities. The overall rate of grade 3 toxicity was 34.9%; with a rate of 8.7% in RT only group and 26.2% in the CRT group (P=0.004). Grade 3 diarrhea, skin toxicity and neutropenia were observed in 14.2%, 17.5%, and 7.9%, of patients.

90.4% of patients had a KPS score >80 and 79.4% of patients had an ECOG of 0 or 1. The median Charlson score was 4, ranging from 0 to 8. The occurrence of grade 3 toxicity was not related to patients’ age (< 80 years vs. 80 years and older, P=0.326), KPS score (P=0.90), ECOG score (P=0.38), number of comorbidities (P=0.324), or Charlson score (P=0.729).

### Outcome and prognostic factors

With a median follow-up time of 19 months, the 3-year OS rate was 48.1% for all patients. The 3-year overall survival rates were 81.5%, 30.8% and 13.7% in patients with curative intent treatment, local control and symptom control, respectively.

Three-year OS rate was significantly higher in patients with primary tumor only than in patients with local recurrence or synchronous distant metastases (68.3% vs. 22.3%, P=0.001), while the 3-year OS rates were similar between patients with local recurrence and with synchronous distant metastases (3-year OS: 32.5% in local recurrence group vs. 13.7% in synchronous distant metastases group, P=0.17). Considering the similar outcomes, we scored 6 points in Charlson Comorbidity Index for patients with local recurrence, equal to the score for distant metastases.

Univariate analyses were performed for the entire series to screen potential factors which predict overall survival in elderly patients, including gender, age, disease status, treatment modality, occurrence of early grade 3 toxicity, KPS score, ECOG score and Charlson score. Patients’ age, disease status and Charlson score were found to be potential prognostic factors for OS (Table 2).

**Table 2:** Univariate analyses for outcomes in elderly patients with rectal cancer

**Characteristics**		**No. (n=126)**	**Hazard ratio**	**95% CI**	**P value**
Gender					
	Male	84			
	Female	42	1.14	0.61–2.13	0.685
Disease					
	Primary tumor only	65			
	Local recurrence	33	2.28	1.03–5.05	0.042
	Synchronous Metastasis	28	3.91	1.89–8.09	0.0002
Treatment mode					
	RT	53			
	CRT	73	0.97	0.52–1.81	0.92
Grade 3 toxicity					
	No	82			
	Yes	44	1.48	0.73–3.02	0.281
Continuous variables					
	Age	126	1.13	1.07–1.20	0.00002
	KPS Score	126	0.99	0.95–1.02	0.558
	ECOG Score	126	1.42	0.93–2.17	0.103
	Charlson Score	126	1.21	1.09–1.36	0.001

*abbreviation: RT, Radiotherapy; CRT, Chemoradiotherapy; CI, Confidence Interval.

**Figure 1:**
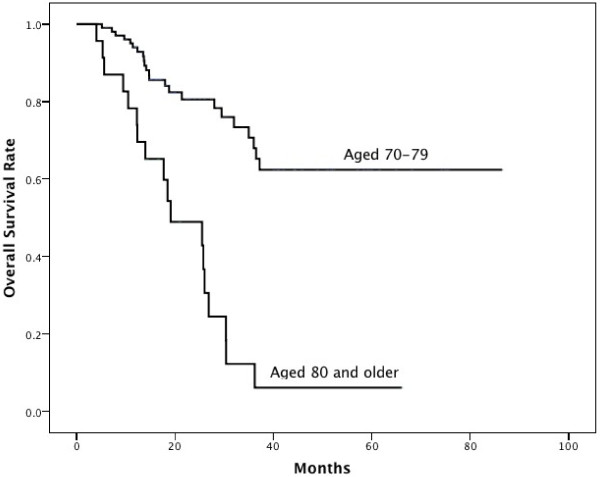
**Difference in overall survival rates of patients aged 70–79 years vs 80 and older.**

**Figure 2:**
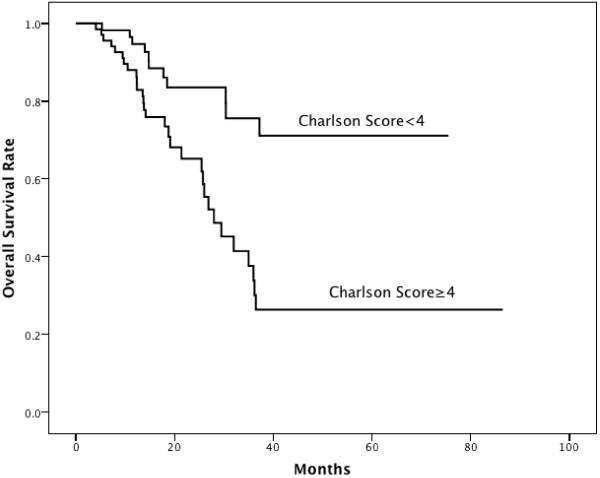
**Difference in overall survival rates of patients with Charlson score <4 vs Charlson score ≥4.**

Multivariate analysis revealed that only age and Charlson score were independent prognostic factors for predicting patients’ 3-year OS. The hazard ratios were 1.12 (95% CI 1.06-1.20, P=0.0001) and 1.20 (95% CI 1.06-1.35, P=0.003), respectively. Subgroup analyses found the 3-year OS rate was 62.4% in patients aged 70–79 years, compared with 6.1% in patients aged 80 years and older (P<0.001, Figure 1). As the median value of Charlson score was 4, we classified patients into two groups (<4 vs. ≥4). The 3-year OS rate was significantly higher in patients with Charlson score <4 than Charlson score ≥4 (71.1% vs. 26.4%, P=0.0003, Figure 2).

## Discussion

Establishing a treatment strategy for elderly patients with rectal cancer is a difficult process. Meta-analysis of randomized studies has shown the benefit of RT in local control regardless of patients' age [7]. However, the improvement in overall survival due to RT is still controversial. Studies from two population based cohorts in the Netherlands and the Dutch Trial showed that patients aged 75 years or older had significantly lower overall survival and higher mortality within 6 months after treatment than patients younger than 75 years. Meanwhile, no survival improvement was observed with the introduction of standard preoperative RT/CRT and surgical resection in patients more than 75 years old [7]. It is important for clinicians to select optimal treatment modalities in elderly patients with rectal cancer by careful assessment of patients' outcomes, treatment risks, life expectancy and comorbidities. Higher rates of surgical complications, more prevalent co-morbidities, and poorer performance status limit the standard use of multidisciplinary treatment in older patients, and treatment deviation is higher in elderly patients than younger patients [8-10]. Margalit et al. reported an 83% rate of early treatment termination, treatment interruption, or dose reduction in patients aged 75 years and older [11]. Aparicio et al. also reported only 37.5% (9 out of 24) of patients aged 75 years and older with locally advanced rectal cancer received RT after surgery [12]. In our series of patients treated with RT or CRT, dose reductions of RT and/or chemotherapy were observed in 31% and 37.3% of patients. Although none of these patients had grade 4 toxicities, almost one-third of patients had grade 3 toxicities, which may be the main factor causing treatment dose reduction. Careful monitoring and frequent modification of treatment were necessary when RT or CRT was used in patients aged 70 or older with rectal cancer. Our study provides useful insight into the tolerability of RT or CRT in a variety of elderly patients with rectal cancer, including locally advanced disease, local recurrence and synchronous metastases.

Performance status is an attempt to quantify cancer patients' general well being and activities of daily life. Two kinds of performance status scale system, the Karnofsky score and ECOG score, are widely used in randomized clinical trials to evaluate patients’ pre-treatment status or measure quality of life. However, most patients were had relative good performance status and fewer comorbidities when receiving cancer-specific treatment. It is difficult to evaluate the value of performance status or comorbidities in predicting treatment toxicity. Currently, performance status scales are mainly used to determine whether patients are eligible for initiating a certain treatment. It had been reported that the use of adjuvant chemotherapy for patients with stage III colon cancer markedly decreased with rising age and comorbidity [13]. In this study, there was no association between patients' performance status or comorbidity and treatment toxicities. The cause may be sub-standard dosage of chemotherapy or RT during treatment, which may minimize the occurrence of grade 3 toxicities.

Currently, the data on combined therapies for rectal cancer used in elderly patients are limited. From analysis of the SEER registry data for a large number of patients, the cancer-specific survival rates in rectal cancer decrease as patient age increases. Oncologically, the decreased use of any cancer directed treatment, an increased use of local treatment, and a decreased use of radical surgery as the patients’ age increased may be attributed to this [14]. However, the SEER registry data cannot be used to evaluate the impact of comorbidities on survival of elderly patients with rectal cancer. Population-based studies have demonstrated that increased age and comorbidity were important prognostic factors for the survival of cancer patients [15,16]. In our study, a significantly decreased of 3-year survival was also observed in patients aged 80 or older. This negative influence of comorbidity on survival of cancer might be due to the increased risk of death due to the comorbid conditions, more contraindications for anti-cancer treatment, more indications for dose reduction and a higher rate of treatment-related complications (infections and cardiovascular events). Other studies have found the effect of comorbidity to be independent of treatment; so sub-standard treatment may not fully account for shorter survival in patients with comorbidities [13,17,18].

The Charlson comorbidity index is a method of predicting mortality by classifying or weighting comorbid conditions (comorbidities) [5]. It has been widely utilized and validated by health researchers for its ability to predict mortality in various disease subgroups, including cancer, renal disease, stroke, intensive care, and liver disease [19-21]. However, the prognostic value of Charlson score in combined treatment of rectal cancer is still unclear. Higher Charlson score was found to be an independent risk factor for poor overall survival in patients with colorectal cancer [12], while other studies did not find a survival difference [22,23]. Charlson score has been used in many studies after modification by neglecting the score of baseline disease like cancer [12,22]. However, since a variety of patients with different stages of rectal cancer were studied in our series, it was reasonable to incorporate the score of solid cancer (scoring 2 for solid cancer and 6 for metastasis) into the score of concomitant comorbidity. Multivariate analysis demonstrated the value of age and Charlson score in independently predicting patients overall survival in stage I-IV rectal cancers. Significantly decreased overall survival was observed in patients with Charlson score≥4. As we incorporated tumor stage into Charlson score, our study provides a simple method to evaluate the outcomes of patients aged 70 or older with varied stages of rectal cancer.

It should be mentioned that we did not perform a relapse-free survival or a cancer specific survival because information on relapse was not clearly obtained in this retrospective study. Some studies have shown that performance status and co-morbidity are both independent prognostic factors [24,25], which therefore may both need to be included in future prognostic studies. Additional factors beside comorbidity may better predict treatment deviation in elderly patients. The International Society for Geriatric Oncology advocates the use of a comprehensive geriatric assessment (CGA) tool to identify baseline factors that may influence treatment tolerability [26]. Studies have shown the ability of CGA in predicting the risk of chemotherapy toxicity and cancer treatment-related morbidity and mortality [27,28]. Otherwise, our study was a retrospective study with a small volume of patients and various treatment regimens; it was difficult to clearly conclude the importance of a certain prognostic factor to select treatment regimens. However, our study addressed an important question regarding the toxicities of RT for elderly patients with rectal cancer, and we also provide a preliminary insight to integrate co-morbidity scoring system to survival analysis in elderly patients. Further randomized clinical trials were needed for these patients.

In conclusion, although the toxicity of RT or CRT was high in patients aged 70 and older, RT or CRT can be safely delivered with careful monitoring and frequent modification of treatment in elderly patients with rectal cancer of varying stage. Patients' age and Charlson score were independent prognostic factors for survival in patients aged 70 and older. Charlson comorbidity score may be helpful in assessing patients’ outcomes in elderly patients with rectal cancer.

## Competing interests

The authors declare that they have no conflicting interests.

## Authors’ contributions

XC, HW and JP designed the study, analyzed and interpreted the data, and drafted the article. JZ and SC participated the study design and revised the manuscript. JP and GC revised the manuscript and provided important intellectual content. HW participated in the acquisition and analysis of data. ZZ participated in the study design, interpreting the data, and was responsible for final approval of the manuscript. All authors have read and approved the final manuscript.
